# How to do research and be a researcher

**DOI:** 10.1192/j.eurpsy.2025.265

**Published:** 2025-08-26

**Authors:** R. Stewart

**Affiliations:** King’s College London, London, United Kingdom

## Abstract

**Abstract:**

There’s a lot of advice and written material available on research methods but much less on **‘**how to be a researcher’. However, understanding this is as much a part of carrying out research as understanding a particular methodology. Having delivered countless teaching sessions on research design, I thought it would be interesting to consider (and attempt to write about) the origins of research principles, how these have developed over time, and what’s common across different fields. As well as this, there are the more mundane realities of attracting funding and publishing output, and the common challenges of achieving career progression and success in complicated political structures. There are great opportunities in research, and few careers that are as continuously interesting and engaging, but it’s wise to keep as clear a perspective as possible of the road behind and ahead.

**Disclosure of Interest:**

R. Stewart Paid Instructor of: Oxford University Press

